# Network-based proteomic approaches reveal the neurodegenerative, neuroprotective and pain-related mechanisms involved after retrograde axonal damage

**DOI:** 10.1038/srep09185

**Published:** 2015-03-18

**Authors:** Caty Casas, Laura Isus, Mireia Herrando-Grabulosa, Francesco M. Mancuso, Eva Borrás, Eduardo Sabidó, Joaquim Forés, Patrick Aloy

**Affiliations:** 1 Group of Neuroplasticity and Regeneration, Institut de Neurociències and Department of Cell Biology, Physiology and Immunology, Universitat Autònoma de Barcelona, and Centro de Investigación Biomédica en Red sobre Enfermedades Neurodegenerativas (CIBERNED), 08193 Bellaterra, Barcelona, Spain; 2 Joint IRB-BSC-CRG Program in Computational Biology. Institute for Research in Biomedicine (IRB Barcelona), 08028 Barcelona, Catalonia, Spain; 3 Institució Catalana de Recerca i Estudis Avançats (ICREA), 08010 Barcelona, Catalonia, Spain; 4 Proteomic Unit, Centre for Genomic Regulation (CRG) and UPF, Dr. Aiguader 88, 08003 Barcelona, Spain; 5 Hand and Peripheral Nerve Unit, Hospital Clínic i Provincial, Universitat de Barcelona, Barcelona, Spain

## Abstract

Neurodegenerative processes are preceded by neuronal dysfunction and synaptic disconnection. Disconnection between spinal motoneuron (MN) soma and synaptic target leads either to a retrograde degenerative process or to a regenerative reaction, depending injury proximity among other factors. Distinguished key events associated with one or other processes may give some clues towards new therapeutical approaches based on boosting endogenous neuroprotective mechanisms. Root mechanical traction leads to retrograde MN degeneration, but share common initial molecular mechanisms with a regenerative process triggered by distal axotomy and suture. By 7 days post-injury, key molecular events starts to diverge and sign apart each destiny. We used comparative unbiased proteomics to define these signatures, coupled to a novel network-based analysis to get biological meaning. The procedure implicated the previous generation of combined topological information from manual curated 19 associated biological processes to be contrasted with the proteomic list using gene enrichment analysis tools. The novel and unexpected results suggested that motoneurodegeneration is better explained mainly by the concomitant triggering of anoikis, anti-apoptotic and neuropathic-pain related programs. In contrast, the endogenous neuroprotective mechanisms engaged after distal axotomy included specifically rather anti-anoikis and selective autophagy. Validated protein-nodes and processes are highlighted across discussion.

Axonal injuries often result in a permanent loss of vital neuronal functions, which occur through a retrograde process of neuronal atrophy and death. This phenomenon is observed in apparently divergent cases such as neurodegenerative diseases, where the axon of an unhealthy neuron degenerates over months before succumb, or after acute peripheral nerve diseases or trauma. In particular, traumatic axotomy or mechanical traction (avulsion) of the nerves is caused by traffic accidents or work and sportive activities, or as a consequence of obstetric complications, which cause severe irreversible dysfunction and/or monoplegia^1^. These lesions are complex to reconstitute surgically due to the absence of a proximal nerve stump by retraction of it ends. When successful reimplantation is performed rerouting other nerves minor recovery of function is obtained^2^,^3^. Root avulsion also commonly results in early deafferentation pain which can develop into central sensitization and severe neuropathic pain. This painful state is usually refractory to pharmacotherapy. Spinal cord dorsal horn central sensitization is linked to neuronal hyperexcitability and the increase of glial immunoreactivity^4^. How acute injury transforms to chronic pain remains a long-standing unresolved question, with important medical implications. Experimentally we can reproduce traumatic root avulsion leading to a time-dependent course of around 80% of motoneuron (MN) loss along the first month after injury^5^. Repeatedly, it has been reported that retrograde degeneration depends on several factors such as age, region of injury (cervical vs lumbar), species of animals examined (rat *vs* mouse) and the distance of the axon injury to the soma. The effects are more severe if the axotomy occurs closer to the soma such as after root avulsion (RA model). In contrast, a distal nerve axotomy plus suture result in MN survival and axonal regeneration (distal axotomy (DA) model,)^6^.Despite recent advances, the molecular mechanisms leading to retrograde MN degeneration are far from clear. Adult MNs do not seem to die by apoptosis as neonatal neurons do after root avulsion. However, this is still a matter of controversy and other authors postulate they may die by necrosis^7^,^8^,^9^,^10^,^11^ In the last years, in order to shed light into this neurodegenerative process, we have collected growing evidences showing that RA and DA triggered common initial molecular events that start to diverge from 7 days post injury (dpo). These differing events are characterized by the unbalanced response of pro-survival mechanisms such as the response to unfolded proteins or autophagy^11^. However the questions of how MNs die and by which mechanisms remain unclear. In addition, no clue has been retrieved about the endogenous mechanism triggered by distal axotomized MNs to allow them survive. In this manuscript, we present a network-based proteome-wide analysis designed to unveil these mechanisms whose relevance for therapy in trauma is also discussed.

## Results

We initially performed a label free proteomic analysis that allows finding out both quantitative and qualitative differences in the rat models of RA and DA performed on the sciatic nerve. Seven days post injury was the targeted time-point where signalling mechanism started to diverge in both models signing for different end course towards either regeneration or degeneration^11^. The LC-MS/MS analysis of the cytosolic fractions from L4–L5 spinal cord segments resulted in the identification of a total of 1,420 proteins with at least two peptides per protein (supplementary Table S1A). A total of 439 and 481 proteins or peptides were significantly altered due to DA or RA respect to the control (p < 0.05) (supplementary Tables S1B and S1C, respectively). In order to dissect out the neurodegenerative processes from the endogenous neuroprotective programs that can be triggered simultaneously, we compared both types on injuries between them and we found that among 567 different proteins (supplementary Table S2A), 109 were found unique to be altered only after DA while 148 signed the RA process (supplementary Tables S2B and S2C, respectively). We attempted for functional annotation of these unique proteins using the DAVID annotation tool, which allowed identifying the most significant biological functions in the data set (FDR < 0.05). In particular for the regenerative course, the cellular components were linked to: chaperoning containing t-complex (66% of enrichment) and SNARE complex (27%) as well as to microtubule cytoskeleton (4.3%) and neuronal projection (3.9%). In contrast, unique proteins in the degenerative process were related to the ribosomal (41%) and translation machinery (41%). Both models shared in common that the most represented biological pathways were linked to that of ribosomes (KEGG#ko03010) (16 proteins involved out of 73). Interestingly, manual curator analysis revealed that 15 proteins among these signatures were enzymes related to aminoacid, fatty acid or citrate cycle metabolisms such as dihydrolipoamide s-succinyltransferase, fatty acid synthase, or malate dehydrogensase, respectively.

Although informative, Gene ontology (GO) terms and KEGG pathways enrichment analyses were difficult to interpret from a patho-physiological perspective since they define general molecular processes that are common to many other disorders. Thus, we developed a computational analysis workflow for interpreting global protein abundance based on the generation of curated biological networks associated to axon injuries and a posterior GSEA analysis. An initial list of key proteins (seeds) associated to molecular processes that could be involved in neuronal response to axon damage was extracted by literature scrutiny, with high content on pathways and protein complexes linked to either neuroprotection/regeneration or degeneration/pain. We then built the networks related to each of these molecular processes directly associated to axonal injury and ran a GSEA analysis to determine whether the expression of genes associated to the motives are significantly altered in any of the conditions.

**Figure 1:**
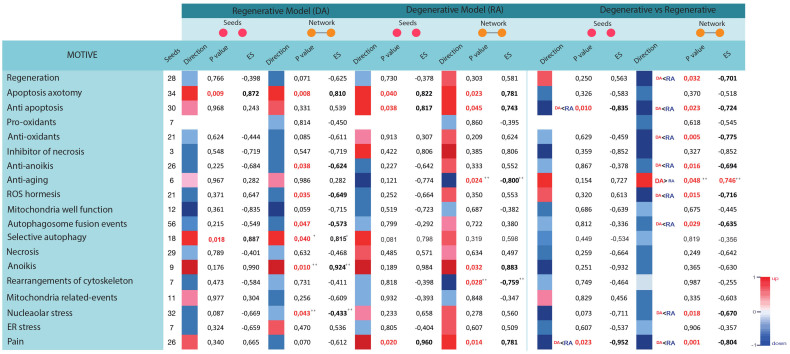
GSEA results of networks related to several molecular processes related to neuronal response to axon damage. Columns indicate the number of key proteins used as seeds to generate networks. The rest of the columns show the results obtain when comparing DA model *vs* control (columns from 2–7), RA *vs* control (from 8–13) or DA *vs* RA (from14–20). Direction column indicates whether the motive is up or down regulated regarding significant differences depending on the p-value when comparing the primary resultant network obtained from seeds (seeds) or that resultant when including also first degree of interactors (network). Enrichment score (ES) is also indicated.

We found that some motives were commonly up or down regulated in both models such as apoptosis and anoikis, a type of programmed cell death with some common pathways with apoptosis. GSEA revealed that genes involved in pathways related to apoptosis were significantly up regulated at protein level in both DA (p value: 0.009) and RA (p value: 0.04) models, in comparison with controls, for seeds, high confidence (first and second degree) and in binary networks (first and second degree). Regarding anoikis, while for DA model only the last type of network was significant (p value: 0.01), for the RA model it was significant for high confidence second degree network (p value: 0.032) and ALL network (p value: 0.021) (Fig. 1).

Some other highly divergent events appear to be associated particularly only to one of the models. On one hand, the RA showed a significant enrichment for anti-apoptosis and pain motives against the seed lists but none of them was significantly enriched in DA models. Pain motive was significantly up regulated in the RA model for seeds (p value: 0.02), high confidence networks (first p value: 0.009 and second degree p value: 0.014) and binary networks (first p value: 0.001 and second degree p value: 0.002). Similarly, anti-apoptosis mechanisms were significantly up regulated for seeds (p value: 0.038), high confidence networks (first p value: 0.025 and second degree p value: 0.045), binary and second degree networks (p value: 0.016) and networks containing ALL interactions (p value: 0.013) (Fig. 1). No more mechanisms were revealed when analysed the seed list, however when considered ALL networks the RA model was significantly enriched for anti-ageing and molecular mechanisms related with the rearrangement of cytoskeleton and organelles motives (anti-ageing p value: 0.024, and rearrangement of cytoskeleton p value: 0.028.).

On the other hand, the DA was significantly enriched for five motives, in comparison with controls, that were not significant in the RA. In particular, selective autophagy motive was significantly up regulated for the seeds (p value: 0.018), high confidence one degree (p value: 0.040) and binary one degree networks (p value: 0.038) whereas anti-anoikis, ROS hormesis, autophagosome membrane fusion, and nucleolar stress events were significantly down regulated (Fig. 1).

The last condition analysed was the direct comparison between DA against RA models. From the direct comparison of the two models, only one motive was up regulated in the DA compared with the RA. The anti-aging motive was significantly up regulated for the ALL network (p value: 0.048). In the other site, five motives were found to be down-regulated in DA models in comparison with RA, such as regeneration, anti-oxidant mechanisms, anti-anoikis, autophagosome membrane fusion events and nucleolar stress. All of them were significant when analysing high confidence first and second degree networks and ALL networks but never when analysing seeds. Finally, anti-apoptosis and pain a well-known physiological process related with neurodegeneration, were also down regulated in the regenerative model when analysing seeds and networks.

**Figure 2:**
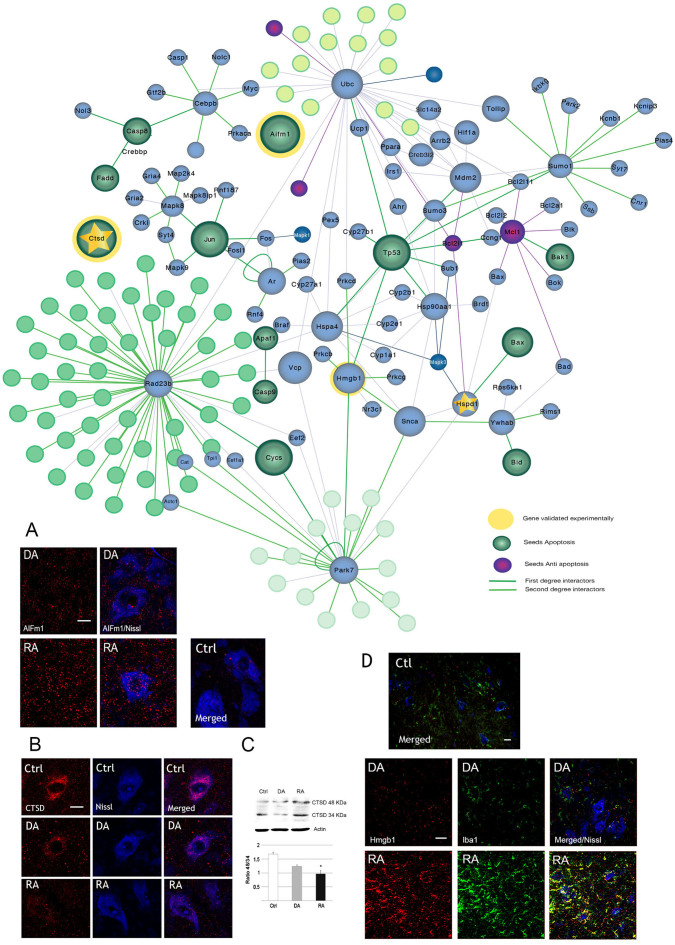
Network topology and validation for the Apoptotic motive *vs* Necrotic motive. Top, Nodes represent proteins and edges represent protein-protein interactions. Green, Purple and Blue seeds belong to the initial motive for which they were previously defined. Dark green edges correspond to direct interactors (first neighbours) and in light green to the second interactors. Squared frame are seeds also found significantly different altered in the proteomic results (control *vs* RA). CTSD and AIFm1 were chosen for further validation in the Apoptotic motive. Bottom panels, (A). representative microphotographies of L4 spinal cord ventral horn showing localization of AIFm1 (red, alone in left columns) within Nissl labelled MNs (blue, merged right columns) from either DA or RA models or control (Ctrl) animals. Note different abundance but no obvious nuclearization of AIFm1 after RA. (B). Localization and abundance of CTSD (red, left) within MNs (blue) in control (Ctrl), RA and DA models. (C). Immunoblot of actin and the immature (48 kDa) or mature (34 kDa) forms of CTSD in the different models and bar graph representing the mean average +/− SEM of the ratio between two CTSD isoforms for each condition (* p < 0.05). (D) Necrosis-related marker in the corresponding network, Hmgb1, is abundantly found outside neurons but co-localizes with microglia (Iba1, green) in both models and contralateral side (Ctl). Scale bar: 20 μm (A) or 50 μm (D).

Overall, these results suggested that some common events co-exist in both models, but others clearly despair allowing the dissection of mechanisms that defined the endogenous mechanisms of neuroprotection *versus* those that prime to neurodegeneration. The resultant motive-associated networks were further analysing in order to choice some key molecules to confirm and help in the interpretation of the GSEA analysis. The results of apoptosis motive was intriguingly appearing significative up-regulated in both RA and DA models and being still a matter of controversy in the literature in the RA model. In the past, we had previously reported the lack of any end-step of caspase 3-dependent apoptosis^11^. So, we search for markers in the network either related to caspase-independent processes such as the apoptosis-inducing factor (AIF), or to initial steps of apoptosis such as Cathepsin D (CTSD) (Fig. 2). AIF is normally present in mitochondria but translocate to the nucleus in caspase-independent apoptotic-related events^12^. We found its presence out of the nuclei of MNs at any condition although an apparent increase in general was observed in the RA model compared to the DA one (Fig. 2A). Thus intermediate apoptotic markers can be recruited and mobilized or altered that indicated somehow that the program is activated but this that no means that the final apoptotic outcome is achieved. Similarly, CTSD is the major aspartyl protease of lysosomes that can be released from them to the cytosol when apoptosis occurs. We observed that it appeared as a dot-like abundant staining in controls but markedly reduced in both RA and DA models with no apparent increase in cytosolic dump in MNs by immunohistochemistry (Fig. 2B). Besides, we performed immunoblotting analysis to identify the different isoforms of CTSD (immature, unprocessed 48-kDa form, *vs* matured 38-KDa form) and we detected a deficiency in processing after RA (Fig. 2C). Thus, apoptotic-related markers were found altered in both models although this do not implicated the existence of executive apoptosis.

Furthermore, we wanted to validate also no-significance in GSEA exploration by studying the expression of necrosis-related maker present in the network such as the chromatin protein high-mobility group B1 (HMGB1). If necrosis exists, abundant HMGB1 should be detected out of the neurons due to membrane bleeding^13^. But it is also reported to be abundantly expressed in activated microglia^14^. By immunohistochemical analysis we observed that abundant HMGB1 accumulated within and outside neurons, in the spinal cord of RA animals compared to DA or control samples. However, the analysis of co-labelling with a widely accepted marker for microglia, Iba1, revealed that HMGB1 was present within microglia instead of spread out throughout the parenchyma of extra-neuronal environment (Fig. 2D). Hence, no necrosis-like phenomenon was observed confirming the prediction by GSEA analysis.

**Figure 3:**
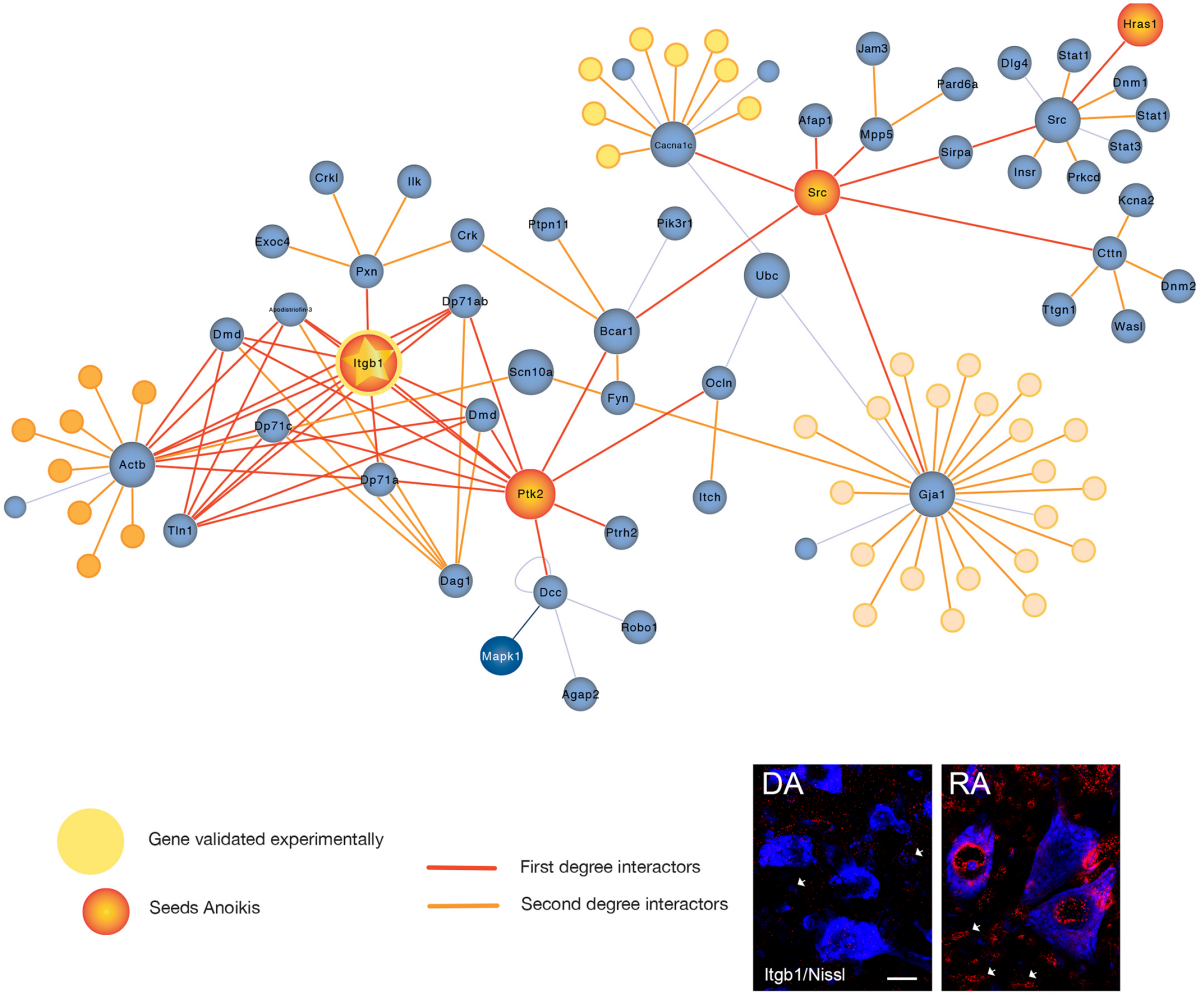
Network topology and validation data for the anoikis motive. Top, Orange and Blue seeds belong to the initial motive for which they were defined previously either anoikis or pain, respectively. Red edges correspond to direct interactors (first neighbours) and in orange to the second interactors. Squared frame are seeds also found significantly different modulated in the proteomic results (control vs RA). Bottom panel, representative merged microphotographies for comparative localization of Itgb1(red) within MNs (blue nissl) in RA and DA models. Differences of itgb1 abundance in surrounding glia are also pointed (arrowheads). Scale bar: 20 μm.

We found anoikis, another program of cell death that is also related to apoptosis to be up-regulated in both models as well. Anoikis is defined as a death process that is induced by inadequate or inappropriate cell–matrix interactions^15^. We chose the b1 subunit of integrins (Itgb1) as a marker presented in the network and up-regulated in the proteomic lists. Itgb1 is normally present in the membrane and we observed this pattern in the DA model (Fig. 3). In contrast, in the RA model we observed a high content of Itgb1 within the cytoplasm or in a perinuclear staining within the MNs (Fig. 3). Since, aberrant retention of Itgb1 within the cells can lead to cell death^16^ the results pointed to anoikis as a relevant process in MN degeneration.

**Figure 4:**
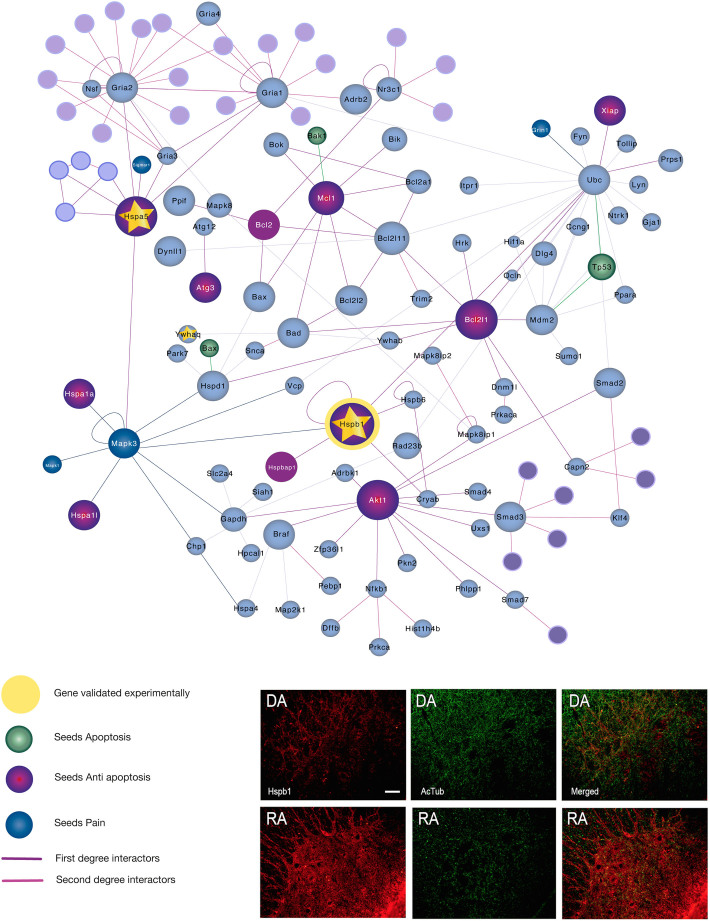
Network topology and validation data for the anti-apoptosis motive. Top, Green and Purple seeds belong to the initial motive for which they were defined previously either apoptosis or anti-apoptosis, respectively. Squared frame are seeds also found significantly different modulated in the proteomic results (control vs RA). Bottom, Immunohistochemical analysis of Hspb1 (red), a chaperone chosen as a target for validation of the motive, and acetylated alpha tubulin (green), one of their substrates, in the ventral horn of the spinal cord from DA and RA models. Scale bar: 100 μm.

On the other hand, in the degenerative process after RA, GSEA analysis revealed the existence of anti-apoptotic process. To validate that, we chose Heat shock protein 27 (Hspb1 or Hsp27) as a marker since it is a chaperone claimed to have major role mediating survival responses functioning as an antioxidant and inhibiting cell death pathways to a range of central nervous system insults^17^. We observed that Hspb1 was presented spread throughout the processes at the spinal cord but their levels were higher in RA samples than in DA as observed by immunohistochemistry (Fig. 4). In contrast, the pattern of acetylated tubulin to which Hspb1 binds was observed altered following the opposite profile. Thus in spite of increased expression of anti-apoptotic markers such as Hspb1, unbalanced ratio of chaperone/substrate may not help to effective survival of MNs.

**Figure 5:**
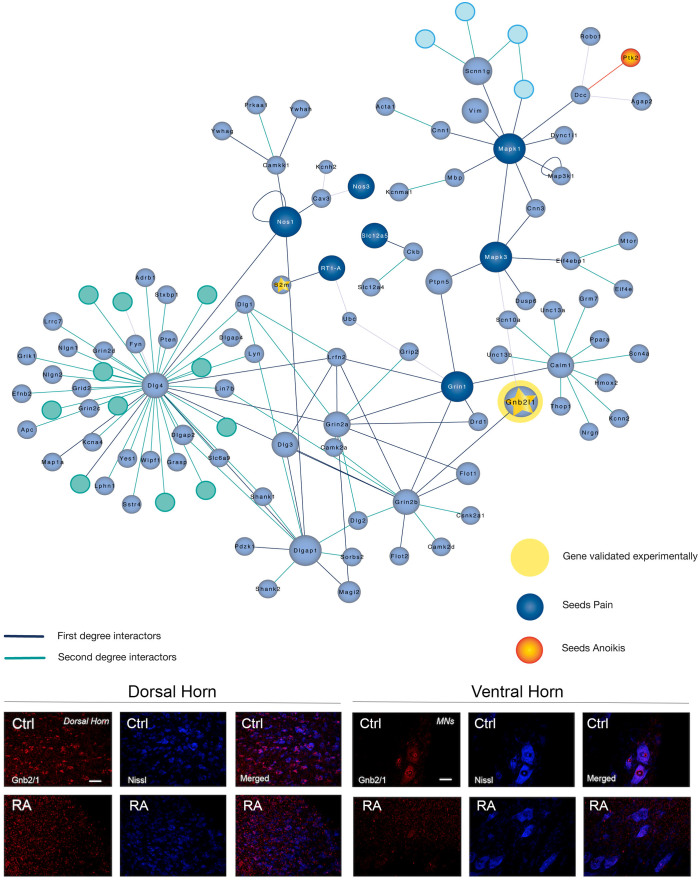
Network topology and validation data for the Pain motive. Top, Orange and Blue seeds belong to the initial motive for which they were defined previously either anoikis or pain, respectively. Validation was performed by immunohistochemical analysis of Gnb2/1 (red) in both dorsal neurons (layers I–III, left panels) or ventral horn MNs (right panels) (blue Nissl) from DA or RA tissue. Scale bar: 100 μm (left, dorsal horn); 50 μm (right, ventral horn).

We finally, analysed a marker related to neuropathic pain-associated network (Fig. 5). It is generally believed that neuropathic pain is an expression of neural plasticity, which can occur at several points of the circuitry including the nociceptive neurons in the dorsal spinal cord. The Receptor for Activated C Kinase 1 (Rack1) is a multifaceted scaffold protein that interacts with the ribosomal machinery, with several cell surface receptors and with proteins in the nucleus^18^. We analysed Rack1 intracellular localization in the dorsal horn of the spinal cord were nociceptive neurons are placed by immunohistochemistry. We found that Rack1, normally placed in the nucleus of the dorsal neurons in control animals markedly changed its subcellular localization to a more diffuse pattern, particularly notably in the RA model (Fig. 5). The meaning for that change and its implication in central sensitization will deserved further analysis as well as other highlighted molecules for this pain associated-network.

**Figure 6:**
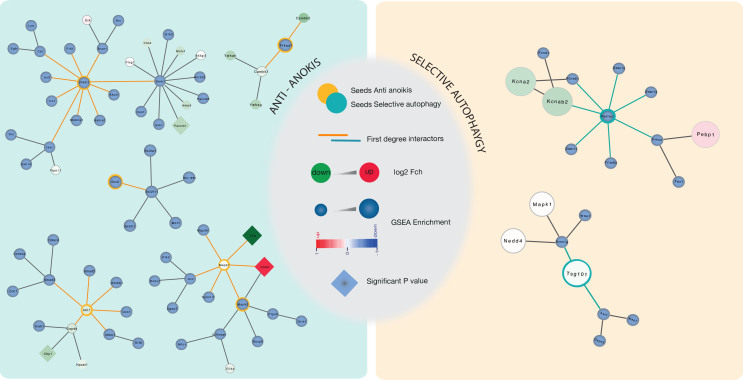
Network topology for the anti-anoikis and selective autophagy motives. Yellow and Blue rings are seeds belonging to the initial motive for which they were defined previously either anti-anoikis or selective autophagy. Light green rounds are proteins form the proteomic list significantly down-regulated (control vs DA). Squared frames indicated different regulated seeds also found significantly different modulated in the proteomic results.

Finally, figure 6 shows the networks associated with anti-anoikis and selective autophagy motives that were significative for the DA model. Several proteins highlighted in green were found significantly altered in the proteomic results compared to controls such as Pacsin1, also known as syndapin 1, the intermediate filament vimentin and the Calcineurin B homologous protein 1 (Chp1) involved in vesicle trafficking. Vimentin (Vim) and acidic calponin (Cnn3) were highly interconnected protein in the network and also highly de-regulated in the proteomic lists. In contrast in the selective autophagy network, there was a lack of key proteins significantly altered in the proteomic lists, although join tendencies revealed the importance of this network as well.

## Discussion

Network-based approaches leverage the idea that complex diseases can be better understood from a more global perspective by looking at pre-defined motives instead of individual genes. In this work we have shown how combining proteomic data with topological information and posterior computational analyses can help us to contextualize our findings and may provide new molecular mechanisms that rely unexplored. We generated 19 biological processes comprising the different molecular events involved in retrograde motoneurodegeneration and neuroprotection after axonal damage and combined them to an unbiased proteomic dataset for posterior GSEA analysis. Although some motives were found enriched for both processes, others were specifically associated with one of them. Peripheral nerve root avulsion that leads to motoneurodegeneration is better explained by pain-related molecular events as well as anoikis, apoptosis, anti-apoptosis and rearrangement of cytoskeleton-related programs. In contrast, the distal axotomy procedure that engages the endogenous neuroprotective process, although including also anoikis and apoptosis, is renowned mainly by the presence of anti-anoikis, ROS hormesis and selective autophagic subroutines. We focus our discussion on some related nodes that are highlighted in each of these significative motives and their possible imbrication to better explain each of these mechanisms. The details of these ineffective and effective packages for neuroprotection pave the way for experimentally targeting better therapeutic strategies for future clinical applications.

Previous attempts to identify and characterize key proteins in neurodegenerative processes that can be targeted for therapy have relied heavily on the observation of up- or down-regulation of individual candidates to estimate effects on neuronal survival. The idea of an unbiased search for proteins differently regulated as a result of a neurodegenerative process is not new, even using the same model of root avulsion presented here in, but those studies were relied only in simple microarray analysis^19^,^20^. The results from the present proteomic study are more than complementary to transcriptome investigations leading to the fact that protein abundance is affected by both the synthesis and turnover and generally cannot be predicted merely from mRNA levels^21^. Besides, our screen has been performed using label free quantitative proteomics which has been demonstrated to be a superior method respect to proteome coverage compared to other labelled-based approaches. However, quantitative results may not reflect strictly differences in relative abundance of a single protein since post-transductional modifications may hinder tryptic digestion and raise quantitative differences in the number of some peptides affected by these modifications yielding qualitative differences. Thus, although results from GSEA analysis indicated some up or down-regulation in the motives, since they are based on altered proteins (either due to abundance or post-translational modifications), attributes in the direction of motives should be considered with precaution. For instance, this was observed for CTSD since by immunohistochemistry we confirmed the existence of different regulation between DA and RA although the opposite direction of that predicted by the proteomic analysis. This apparent contradiction may be due to the observed different processing of the protein in both models which was less efficient in the RA model. This demonstrates that different peptide detection of different isoforms of the same protein can lead to an unspecific read out of up/down regulation. So, the interpretation of the results deserve careful analysis of an specific protein than merely look for over/down expression as done in transcriptomic studies. This feature of the dataset forced to use a more sophisticated computational analysis to search for biological meaning. The main benefits of analysing phenotypic and proteomic differences in diseases in a network context is that by mapping our seeds to an interaction network and searching for GSEA we are increasing the statistical power of our analyses. Biological meaning was better achieved through GSEA analysis than by general GO terms and KEGG pathways analysis commonly used to get functional enrichment in many other cases. Moreover, including in our analyses the direct interactors of our seeds we found significantly enriched motives not so when analysing seeds alone, providing new candidate genes potentially associated with our model or unveiling unknown mechanisms of action. This was the case of the anti-anoikis motive which was significant only when direct interactors were included in the signature for the DA model. Thus, our methodological approach shed more accurate mechanistic information than any other previously reported transcriptome/proteome analysis.

Regarding the mechanisms discovered, as expected, those triggered after root avulsion involved pain-related motives. Although our RA model is not considered a classical model to study neuropathic pain, recently it has been characterized the presence of nociceptive alterations^22^. Accordingly, we have revealed a particular network that may help explaining the appearance of central sensitization in this model. The network includes several already described molecules implicated in pain apparition in other models studied such as dlg4 or PSD-95, NOS1-3, linked to both inflammatory and neuropathic pain, and Grin2a–2b corresponding to NMDA receptors, NR2A and NR2B, respectively, which overactivation favours the hyperexcitability of the nociceptive circuit^23^. Novel relationship that appears from our analysis is related to pain and MAPK3, commonly associated to regeneration in the literature^24^. We have found in the middle, bridging NMDA and MAPK3, the protein Grbl/2 or receptor for activated C kinase 1 (RACK1). Several reports have demonstrated its involvement in multiple biochemical pathways; however, its implication in pain sensitization has not been yet connected. RACK1 binds to ribosomes, Src kinases and/or Protein kinase C simultaneously, and it inhibits the Src kinase. It has been reported that activation of Src-Family Protein Tyrosine Kinases in the spinal dorsal horn is essential for the initiation and development of central sensitization after peripheral tissue and nerve injury^25^. Besides, one of its best-characterized substrates is the NR2B subunit whose phosphorylation triggered downstream signalling transduction^26^, and causes NR2B receptor-dependent neuronal plasticity and pain sensitization^27^. Rack1 is normally located in the nucleus of the cells and 14-3-3 protein (also named Ywhaq) promotes this translocation^28^. We found Rack1 misplaced out of the nucleus in dorsal neurons after damage, observation consistent with the putative down-regulation of Ywhaq after RA at the proteomic list. It is plausible that delocalized Rack1 would release Src to be activated and consequent NMDA increased activity would lead to hyperexcitability. Although still speculative, the triode form by Rack1, Src and NMDA seems to be an interesting pathway to take into consideration for neuropathic pain investigations.

It is intriguing that pain-related network was extremely imbricated within our other networks. For instance, Src appears indeed in the Anoikis sub-routine presented in both models. Anoikis is a cell death modality ignited by detachment from the extracellular matrix (ECM)^29^, whose resistance has been extensively studied in metastatic cells. Almost no studies have been found in the neurodegenerative field in spite of the demonstrated importance of EM attachment for neurons to survive^30^. During development, MNs that do not obtain the correct trophic support due to lack of proper nerve-muscle connections, die by anoikis^31^ but nobody has studied its relevance in adult axotomized MNs. Integrins anchored to plasmatic membrane allow the adhesion to EM and couples to the intracellular actin-cytoskeleton remodelling through FAK-Src complex, to maintain cell survival in adhesive cells. Axotomized MNs might suffer from anoikis and might trigger downstream apoptotic program in both DA and RA models, although with substantial differences. We observed Itgb1, the common subunit in all integrin complexes, abundantly accumulated in the interior of the cell instead of being properly transported to the plasmatic membrane after RA in contrast to DA. MNs in the DA model might initiate detachment to prepare themselves for axon prolongation to regenerate while in RA Itgb1 is futile overexpressed. This might contribute determinately to neuronal death. The existence of anoikis in both models although with very different features is consistent with the engagement of apoptotic programs.

It has been long reported the observation of apoptotic markers in avulsed mouse models such as BAX and p53^32^,^33^ Using a model of avulsion of the entire sciatic nerve, a distal lesion similar to DA but without suture which yield 20% of MN death, Martin et al observed that BAX and p53 KO mice block that and concluded it was an apoptotic type of death^33^. The implication of BAX in the loss of MNs after root avulsion could not be clarify by using adult BAX-KO mice^32^. Besides differences in experimental models which are relevant to steer MNs toward one or other cell death program, it should be taken also into consideration that pleiotropic effect of p53 promote both apoptotic programs (BAX dependent) and inhibition of anti-anoikis programs mediated by AKT^34^ (see below). Thus, from those experiments dual interpretation can be envisaged. From our work, we concluded that engagement of the anti-anoikis program may be the key successful one to boost endogenous neuroprotective process after axonal damage. So, the increased MN survival obtained by blocking p53 can be interpreted by reinforcing anti-anoikis program even when MN death is not apoptotic. We demonstrated that none of the models analysed presented degeneration ended up by apoptosis. This might be thanks to the presence of concurrent anti-apoptotic subroutines that prevents this in the RA model. Indeed, inhibitors X-linked Inhibitor of apoptosis proteins (XIAP) are already higher express in adult versus neonatal MNs and it seems to be key molecules to render adult MNs more resistant to axotomy injuries^35^. We validated this observation analysing the expression of Hspb1 as an anti-apoptotic molecule^36^. Hspb1 is a small multitarget chaperone that binds and influence on the stability of the tubulins. Mutations in hspb1 leading to excessive chaperone activity, have been recently found linked to peripheral neuropathy called Charcot Marie-Tooth disease probably due to microtubule stabilization^37^. Balanced stoichiometry between Hspb1 and acetylated tubulin is key for correct function of the cytoskeleton precluding any pro-survival effect^38^. We found that the ration was inverted after RA. So again, the overexpression of an anti-apoptotic molecule does not guarantee the successful recovery form death. The presence of opposite forces may contribute to slowness of the death process in RA which is definitely ending lacking apoptotic caspase-dependent or independent, executive action. This is interesting since we suggested dissociation between triggering or initiating programs and final outcome that has probably long contribute to the controversy present around this mechanism of neurodegeneration. Avulsed MNs will probably finally die due to a combination of detachment, isolation, and anoikis in addition to internal cytoskeletal blocking of the transport that may difficult resolve properly the programs engaged either towards apoptosis, that needs proper translocation and energy supply, or towards anti-apoptosis. Research should be reinforced to verify whether this corresponds to a new form of death that can be found in vivo in both acute process and in other chronic diseases specifically affecting MNs.

Although out of scope of the present manuscript, proteomic analysis also revealed some particular enzymes involved in amino acid, fatty acid or citrate cycle metabolism that sign the neurodegenerative process. Abnormal concentrations of metabolites that reflect axonal loss or dysfunction have been reported. The most common linked finding in the literature is a reduced concentration of N-acetyl-aspartate^39^. Recently, Kachramanoglou and collaborators reported that increased levels of myo-inositol/creatinine ratio may serve as a marker of avulsion injury^40^. However, this parameter seem to diminish with time cause it might be linked to persistent gliosis. Our present study proportionate information about other putative metabolites that can selectively associated with the neurodegenerative avulsion injury distinguished from a regenerative form. Analysis of this metabolic pathways and deductive subproducts might be of great value for clinical outcome measures in trials of repairing strategies.

Regarding the neuroprotective process, what makes successful the distal axotomy model. One clue may be related to the choice of other neuroprotective routes instead of the anti-apoptotic one. In this regard, we found that anti-anoikis, ROS hormesis and autophagy-related events seem to be the successful choices. Indeed, recent work demonstrated that ECM detachment also robustly induces autophagy to presumably protect against this type of stress^41^. A basal level of autophagy has been shown to have a neuroprotective role, although excess autophagy is harmful^42^,^43^ and the autophagy of ubiquitylated cytosolic proteins is the second mechanism by which ubiquitin may signal protein degradation of selective unwanted proteins in lysosomes. For instance, Src, which is essential for the anoikis response as mentioned, can be entrapped and degraded by autophagosomes leading to cell survival^44^. Selective autophagy can be engaged to manage ubiquitylated cargo captured by the endosomal sorting complex for transport (ESCRT) machinery. ESCRT machinery directly or indirectly, regulates autophagy. By GSEA analysis we found significative enrichment of components of the ESCRT complex such as TSg101, putatively down-regulated in the DA model, and imbricated in the same motive with late-autophagic-related molecules such as p62/Sqstm1, the ubiquitin-binding protein^45^. Thus, these observations suggested that proper running of the ESCRT machinery and autophagy course might be key elements in promoting survival after axonal injury.

Finally, several of the proteins we related to both the anti-anoikis motive and the membrane fusion events were found down-regulated in the DA model such as Pacsin 1, scamp1, a secretory carrier membrane protein, NSF, Dlg4 and chp1 (Calcineurin B homologous protein 1) and SNAP25. All of this proteins are related to vesicle-mediating transport and endocytic/exocytic membrane traffic often involved in axonal growth^46^. These results suggested that proteins involved in axon tip extension might be transported to the lesion site, within the peripheral nerve and far away from the neuronal soma in the spinal cord object of our proteomic analysis. Interestingly, the connections we found between those and phosphatidylinositide 3-kinase (PI3K) in this network deserves special mention since it normally increases in both distal axons and cell bodies because required for both the neurotrophin-induced cell survival and distal axon growth^47^. The role of Pi3K-AKT activation as a survival-promoting factor in injured neurons is well documented^48^ and so we highlighted here its importance in the anti-anoikis program together with selective autophagy as key elements for the endogenous neuroprotective mechanisms.

In conclusion, we have shed some light about the process involved in retrograde MN reactions to axotomy that are delicate balanced and can lead to either degeneration or neuroprotection depending on parameters such as distance of the lesion. The neurodegenerative process is leading to opposite forces, apoptosis, anoikis and anti-apoptotic programs, which may contribute to retard the fatal outcome as well as to give it a characteristic that is not definitely apoptotic, nor necrotic. Further establishment of the molecular parameters that define it may lead to the definition of a new form of neuronal death, not as pure as those observed in vitro and well established by the Nomenclature Committee of Cell Death but certainly present in an *in vivo* process. At present we do not know the incidence or implication of this process in other neurodegenerative processes but we think that this deserves further exploration. On the other hand, we have dissected out those neuroprotective programs that can lead to successful recovery after axotomy that include rather co-running of anti-anoikis and selective autophagy programs. These are relevant for therapeutical approaches. The unsuccessful approaches trying to impede the neurodegenerative process, using anti-apoptotic agents for instance, or any anti-death approach have been demonstrated to be unsuccessful in trauma or in neurodegenerative diseases. So, we show here that the tissue has the capacity to recover them self and knowing how to do it may be key for therapy and an alternative way of thinking for therapeutical strategies.

## Methods

### Models

Sprague–Dawley female rats aged 12 weeks were kept under standard conditions of light and temperature and fed with food and water ad libitum. We performed surgical procedures under anesthesia with a cocktail of ketamine/xylazine 0.1 mL/100 g weight i.p essentially as reported previously^5^. To perform extravertebral nerve root avulsion of the L4–L5 roots we made a midline skin incision to identify each side sciatic nerve and applying a moderate traction on selected roots away from the intervertebral foramina, obtaining the mixed spinal nerves that contained the motor and sensory roots and dorsal root ganglia out. To produce the distal axotomy plus suture model, the sciatic nerve was exposed at mid high and freed from surrounding tissues; then, the nerve was transected and immediately repaired by fascicular suture (10–0, Ethicon). After both types of lesions, the wound was sutured by planes, disinfected with povidone iodine and the animals allowed recovering in a warm environment. All procedures involving animals were carried out in accordance with the guidelines of our institution and experimental protocols were approved by the Ethics Committee of our institution, and following the European Community Council Directive 86/609/EEC.

### Sample preparation

We anesthetized groups of rats (n = 4–5) with root avulsion or distal axotomy and un-operated controls at 7 dpo to obtain L4–L5 spinal cord segments (5-mm length) samples to be snap-frozen into liquid nitrogen. We homogenized the tissue in lysis buffer (Hepes 20 mM, Sucrose 250 mM, EDTA 1 mM, EGTA 1 mM and a cocktail of protease and phosphatase inhibitors) with potter homogenizer on ice. After centrifugation of lysates at 800 × g for 20 min at 4°C, we collected the supernatant as a cytosolic fraction and quantified by BCA assay (Pierce Chemical Co.; Rockford, IL, USA). For proteomic analysis, we solubilized 75 μg of each sample in 4% SDS, 8 M Urea, 0.1 M HEPES, 0.1 M DTT and subsequently reduced, alkylated in 0.05 M iodoacetamide and digested with trypsin (ratio enzyme:substrate 1:10) using the method FASP (Filter Aided Sample Preparation) as described^49^. All samples were treated in parallel.

### Proteomic analysis

We analysed samples using an LTQ-OrbitrapVelos mass spectrometer (Thermo Fisher Scientific) coupled to a ProxeonEasyLC (Thermo Fisher Scientific). We loaded the peptide mixtures directly onto the analytical column (2 μl·min^−1^) and separated them by reversed-phase chromatography using a 15-cm column with an inner diameter of 100 μm, packed with 5 μm C_18_ particles (NikkyoTechnos Co., Ltd.). Chromatographic gradients started at 97% buffer A (0.1% formic acid (FA) in water), and 3% buffer B (acetonitrile, 0.1% FA) with a flow rate of 500 nl·min^−1^, and gradually increased to 85% buffer A + 15% buffer B in 4 min, and to 55% buffer A + 45% buffer B in 120 min. We operated the instrument in TOP20 DDA (Data Dependent Acquisition) mode with one full MS scan in the Orbitrap at a resolution of 60,000, and a mass range of m/z 350–2,000 followed by MSMS spectra of the 20 most intense ions. We utilized the Ion Trap by CID (collision induced dissociation) to produce fragment ion spectra and used normalized collision energy at 35%. We acquired all data with Xcalibur software v2.1. We used Proteome Discoverer software suite (v1.3.0.339, Thermo Fisher Scientific) and the Mascot search engine (v2.3.01, Matrix Science^50^) for peptide identification and quantitation. We analysed the data against SwissProt Rat database (released July 2012) containing the most common contaminants (599 entries). We used a precursor ion mass tolerance of 7 ppm at the MS1 level and allowed up to three miscleavages for trypsin. The fragment ion mass tolerance was set to 0.5 Da. Oxidation of methionine and N-terminal protein acetylation were set as variable modifications whilst cysteine carbamidomethylation was set as fixed modification. We filtered the peptides based on their false discovery rate (FDR > 5% not considered). For peptide quantification, we considered the chromatographic peak of the peptides calculated by Proteome Discoverer, and median normalized the areas by log2 transformation using R 3.0.2. We quantified the data using the R package *MSstats* (v. 2.0.1)^51^,^52^,^53^. For each ratio, we calculated the adjusted p-value^54^ (p < 0.05 for significance). Finally, we performed gene ontology and pathway analysis for regulated proteins with STRING^55^ (http://string-db.org/) and DAVID Web tools^56^ (http://david.niaid.nih.gov/david/version2/index.htm).

### Protein-protein interaction networks

Through manual curation of the literature, we identified the main biological processes (BP) described to be involved in either regenerative or/and degenerative processes and considering all possible events that may occur around 7 dpo after injury. By this time, in the RA model there might happen some problems in autophagy late-events and ER stress canonical response as we have previously suggested^11^. Besides, we wanted to determine whether apoptotic, necrotic or other death-associated pathways such as anoikis^31^, nucleolar stress^57^, and mitochondrial dysfunction^58^ that has been described in other neurodegenerative processes, were also present. In addition, we included a pain-related motive since the apparition of neuropathic pain is markedly relevant after root avulsion^59^. In association with the the DA model, we included a list of motives classically involved in regeneration and some anti-cell death progrmams in order to reveal the endogenous neuroprotective mechanisms in the promotion of neuronal survival and regeneration such as anti-anoikis^60^ or anti-apoptosis and anti-oxidant hormesis^61^. Subsequently, we classified all of them into 19 different motives comprised by altogether 331 different genes (that we named seeds) (Table 1, supplementary Table S3). From those 19 different motives, 10 of them accounted for processes related to neuron regeneration and 9 to neuron degeneration.

For protein interaction data, we collected a subset of all the available Protein-Protein Interactions (PPIs) from an integrated database containing data from nine major public PPIs databases: Intact^62^, MINT^63^, DIP^64^, MatrixDB^65^, InnateDB^66^, BioGRID^67^, BIND^68^, MPIDB^69^, and HPRD^70^, mapping protein identification codes to Uniprot Accession Codes (Uniprot KB version 2013_01). We collected all experimentally verified direct interactions adding only those described as binary according to the associated detection methods^71^. A part from the binary interactome we generated a second interactome by collecting all experimentally determined interactions using the ‘spoke expanded’ model in order to transform protein complexes into binary interactions when the bait of the given affinity purification was specified and the ‘matrix expanded’ model otherwise^72^.

From the previous mentioned interactomes, we constructed two types of networks. First degree networks, which consist of adding the direct interactors of the motive seeds, and second degree networks by adding an extra level of interactors to the first degree networks (i.e. also adding direct interactors of the seed interactors). We assigned a confidence score to each interaction, based on the size and type of experiment and the number of publications supporting the interaction^73^,^74^ and used it to build high-confidence networks by filtering out those interactions with a normalized confidence score below 0.5.

### Gene set enrichment analyses (GSEA)

Gene Set Enrichment Analysis is a computational method that determines whether a predefined list of genes (that we name ‘signatures’) shows significant differences between two biological phenotypes under study (Subramanian, Tamayo et al. 2005). We performed a GSEA using as signatures our lists of biological process-associated genes (one for each motive) and our set of protein-protein interaction networks generated from them. We used GSEA as implemented in the Bioconductor library phenoTest (R package version 1.9.0) to assess if the proportion of genes associated with phenotype in each signature is greater than that outside of the signature. GSEA requires first ranking genes according to their association with a given phenotype (each neurodegenerative process), and then determining whether genes included in the signature tend to present either positive or negative enrichment values. Association with each model was measured using the previously estimated log2 fold changes obtained from our proteomic analysis. The output of the GSEA is an enrichment score (ES), a normalized enrichment score (NES) which accounts for the size of the gene set being tested, a P-value and an estimated False discovery rate. Computing NES, P-values and FDR requires a permutation-based approach for which we computed 10,000 permutations per signature.

### Data validation

We used immunohistochemistry and western blot for data validation. After deeply anesthetized with dolethal we transcardially perfused the animals with a saline solution containing heparin (10 U/ml), followed by 4% paraformaldehyde in a 0.1 M phosphate buffer, pH 7.2, for tissue fixation at 7 dpo (n = 4 at each time post lesion) and removed the L4 and L5 segments (5-mm total length) of the spinal cord, post-fixed in the same fixative for extra 4 h and cryopreserved in 30% sucrose overnight. We cut the samples into serial transverse sections (20-mm thick) onto gelatinized slides using a cryotome (Leica, Heidelberg, Germany) and preserved them at −20°C until used. For immunohistochemistry, we treated the slides with blocking solution in Tris-buffered saline (TBS) with 0.03% Triton-X-100 and 10% bovine serum for 1 h and incubated thereafter with different primary antibodies: Cathepsin D (CTSD), High-Mobility Group Box 1 (HMGB1), Apoptosis-Inducing Factor, Mitochondrion-Associated, 1 (AIFM1), Guanine Nucleotide Binding Protein (G Protein), beta Polypeptide 2-Like 1 (GNB2L1, RACK1), Heat Shock 27 kDa Protein 1 (HSPB1) (Antibodies-online, Aachen, Germany) overnight at 4°C. After several washes with TBS, 0.05% Tween-20, the sections were incubated for 2 h with Cy-2 or Cy-3 conjugated donkey anti-rabbit antibodies (Jackson Immunoresearch, West Grove, PA, USA). We counterstained the sections with DAPI (Sigma, St Louis, MO, USA), or NeuroTrace Fluorescent Nissl Stain (Molecular Probes, Leiden, Netherlands) and mounted the slices with Fluoromount-G mounting medium (SouthernBiotech, Birmingham, AL, USA). Images of the spinal cord samples from different treatments and controls were taken under the same exposure time, sensibility, and resolution for each marker analysed, with the aid of a digital camera (Olympus DP50) attached to the microscope (Olympus BX51). Confocal microscope examinations were performed with a Confocal Laser Scanning Microscope (Zeiss LSM 700; Zeiss, Jena, Germany). For western blotting, we loaded thirty micrograms of cytosolic fractions of L4–L5 segments from each animal model onto 12% SDS-polyacrylamide gels to perform electrophoretic separation of the proteins following by its transference to a PVDF membrane in a BioRad cubette system in 25 mM Tris, 192 mM glycine, 20% (v/v) methanol, pH 8.4. We blocked the membranes with 10% non-fat dry milk in TBS for 1 h at room temperature and then incubated overnight with primary antibody anti cathepsin D (1:1000) or anti-actin (Sigma). After several washes, membranes were incubated for 2 h with an appropriate secondary antibody conjugated with horseradish peroxidase (1:3000) (anti-mouse-HRP, Dako Denmark; Glostrup, Denmark) and anti-rabbit-HRP (Invitrogen Corp.; Carlsbad, CA, USA). The membrane was visualized using a chemoluminiscent mix 1:1 [0.5 M luminol, 79.2 mM p-coumaric acid, 1 M Tris-HCl; pH 8.5] and [8.8 M hydrogen peroxide, 1 M Tris-HCl; pH 8.5], and the images analysed with Gene Snap and Gene Tools softwares, and Gene Genome apparatus (Syngene, Cambridge, UK).

## Supplementary Material

Supplementary Information supplementary table 1

Supplementary Information supplementar table 2

Supplementary Information supplementary table 3

## Figures and Tables

**Table 1:** Motives and number of seed proteins

Biological Processes (Motives):	Number of seeds	References
**Regenerative Process (DA)**		
Regeneration	28	24,75,76
Apoptosis axotomy	34	77
Anti apoptosis	30	61
Pro-oxidants	7	78
Anti-oxidants	21	78
Inhibitor of necrosis	3	77
Anti-anoikis	26	79
Anti-aging	6	61
ROS hormesis	21	61
Mitochondria well function	12	80
**Degenerative Process (RA)**		
Autophagosome fusion events	56	81
Selective autophagy	18	82,83
Necrosis	29	77
Anoikis	9	84
Rearrangements of cytoskeleton & organelles	7	85
Mitochondria related-events	11	58,86
Nucleolar stress	32	87,88
ER stress	7	89,90
Pain	26	91
Total number of seeds	383	92
**Unique seeds**	**311**	
